# Supplementary figures and images for: Innovative Approach to Enhance Bioavailability of Birch Bark Extracts: Novel Method of Oleogel Development Contrasted with Other Dispersed Systems
Source: Plants (Basel). 2024 Jan 4;13(1):145. doi: 10.3390/plants13010145 (PMC10780823; doi:10.3390/plants13010145)

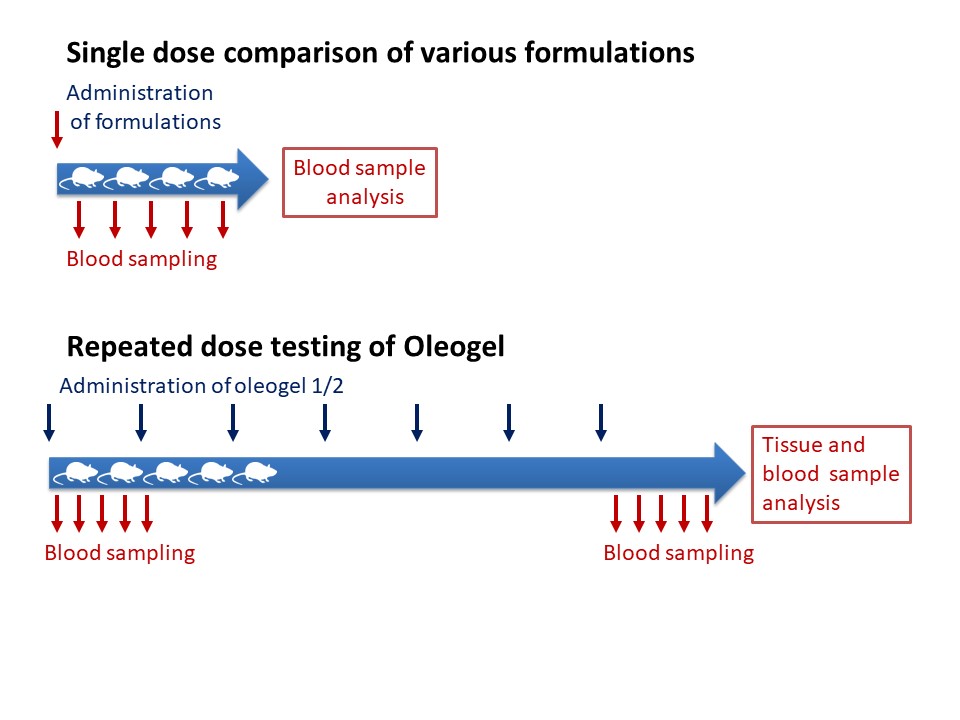

Supplement: Supplementary file 1 [file plants-13-00145-s001.zip › plants-2742379-supplementary.jpg]
